# Increased Intrinsic Connectivity of the Default Mode Network in Temporal Lobe Epilepsy: Evidence from Resting-State MEG Recordings

**DOI:** 10.1371/journal.pone.0128787

**Published:** 2015-06-02

**Authors:** Fu-Jung Hsiao, Hsiang-Yu Yu, Wei-Ta Chen, Shang-Yeong Kwan, Chien Chen, Der-Jen Yen, Chun-Hing Yiu, Yang-Hsin Shih, Yung-Yang Lin

**Affiliations:** 1 Institute of Brain Science, School of Medicine, National Yang-Ming University, Taipei, Taiwan; 2 Department of Neurology, School of Medicine, National Yang-Ming University, Taipei, Taiwan; 3 Institute of Clinical Medicine, School of Medicine, National Yang-Ming University, Taipei, Taiwan; 4 Institute of Physiology, School of Medicine, National Yang-Ming University, Taipei, Taiwan; 5 Brain Research Center, National Yang-Ming University, Taipei, Taiwan; 6 Department of Education and Research, Taipei City Hospital, Taipei, Taiwan; 7 Laboratory of Neurophysiology at Medical Research Division, Taipei Veterans General Hospital, Taipei, Taiwan; 8 Department of Neurology, Taipei Veterans General Hospital, Taipei, Taiwan; 9 Department of Neurosurgery, Taipei Veterans General Hospital, Taipei, Taiwan; Banner Alzheimer's Institute, UNITED STATES

## Abstract

The electrophysiological signature of resting state oscillatory functional connectivity within the default mode network (DMN) during spike-free periods in temporal lobe epilepsy (TLE) remains unclear. Using magnetoencephalographic (MEG) recordings, this study investigated how the connectivity within the DMN was altered in TLE, and we examined the effect of lateralized TLE on functional connectivity. Sixteen medically intractable TLE patients and 22 controls participated in this study. Whole-scalp 306-channel MEG epochs without interictal spikes generated from both MEG and EEG data were analyzed using a minimum norm estimate (MNE) and source-based imaginary coherence analysis. With this processing, we obtained the cortical activation and functional connectivity within the DMN. The functional connectivity was increased between DMN and the right medial temporal (MT) region at the delta band and between DMN and the bilateral anterior cingulate cortex (ACC) regions at the theta band. The functional change was associated with the lateralization of TLE. The right TLE showed enhanced DMN connectivity with the right MT while the left TLE demonstrated increased DMN connectivity with the bilateral MT. There was no lateralization effect of TLE upon the DMN connectivity with ACC. These findings suggest that the resting-state functional connectivity within the DMN is reinforced in temporal lobe epilepsy during spike-free periods. Future studies are needed to examine if the altered functional connectivity can be used as a biomarker for treatment responses, cognitive dysfunction and prognosis in patients with TLE.

## Introduction

Temporal lobe epilepsy (TLE) is the most common type of focal symptomatic or cryptogenic epilepsies, and it is characterized by recurrent, unprovoked seizures originating from the medial or lateral temporal lobe. TLE mainly consists of simple partial seizures (SPS), complex partial seizures (CPS) and secondary generalized tonic-clonic seizures (SGTCS) with or without loss of consciousness during the ictal period. TLE is associated with various cognitive dysfunctions, including intellectual, language, visuospatial and memory dysfunction [1–3].

The default mode network (DMN) in the brain is consistently activated during a resting-state condition that is free from attention demands or cognitive load [4, 5]. Many anatomically distributed and separated cortical regions constitute the DMN, including the posterior cingulate cortex (PCC), precuneus (PCu), inferior parietal cortex (IPC), medial temporal (MT) lobes, medial frontal cortex (MFC), and anterior cingulate cortex (ACC) [4–6]. The DMN is associated with a variety of cognitive functions, such as the theory of mind and social cognition [7], episodic memory [8], emotion and anxiety [9], and low-level attentional focus [10]. Abnormalities in the DMN have been observed in Alzheimer’s disease [11–13], schizophrenia [14], depression and anxiety [15], autism spectrum disorder [16], attention deficit/hyperactivity disorder [17], and many other conditions. Further investigations into the DMN could be a promising approach to unveil the underlying pathological mechanisms of multiple diseases.

In TLE, resting functional MRI studies have explored blood oxygen level-dependent (BOLD) signals in the epileptogenic and DMN regions [18, 19]; in addition, studies have examined the functional connectivity between epileptogenic foci, particularly within medial temporal lobe and brain areas within the DMN with [20, 21] or without [22–26] the simultaneous recording of EEG. Of note, during interictal epileptic discharges, the amplitude of the BOLD signal was increased in the mesial temporal lobe while decreased in the DMN [18, 19]. Moreover, disruption of the functional connectivity between the mesial temporal and DMN regions has been found in TLE [20, 22–26]. These changes in functional connectivity may result from the cognitive or psychiatric impairments associated with TLE. Nevertheless, the epileptic activity from the temporal lobe may spread into functionally interconnected DMN regions [19, 27, 28], which raises the question as to how the DMN works during interictal spike-free periods in TLE. Several recent fMRI studies have begun to assess this question using simultaneously EEG recordings [20, 21]. Notably, Lin et al. [29] reported that simultaneous magnetoencephalography (MEG) and electroencephalography (EEG) recordings improve epileptic spike detection in TLE because of a portion of interictal spikes that are selectively recognized by MEG or EEG techniques.

The cognitive deficits observed in TLE are related to the laterality of seizure onset. The left TLE was reported to be prominent impairment in verbal memory; nevertheless, the right one was impaired in nonverbal memory [30, 31]. However, many studies have found inconsistent findings of intelligence deficits in epilepsy for the factor of lateralization [30, 32, 33]. Since the DMN was found to be associated with cognitive function, two recent fMRI studies have shown the effect of lateralized TLE on the DMN [34, 35]. The distinct alterations in the functional connectivity of the DMN between the left and the right TLE were reported in these two studies; however, there are inconsistencies between the two studies on the extent of cortical involvement that warrants further investigation.

It is well known that fMRI does not directly measure neural activity [36, 37], and its signal analysis is confined to a relatively low temporal frequency. Recent evidence [38–41] has shown that fMRI activation was closely coupled with EEG energy modulation in the gamma and high-frequency range (> 40 Hz). Although fMRI studies have begun to report the presence of DMN alterations in TLE, it is unclear how the electrophysiological signatures of DMN connectivity in TLE relate to frequencies < 40 Hz. This information could help explain the temporal dynamics of ongoing brain activity and provide novel neurophysiological correlates of mental dysfunction. Moreover, a network perspective using oscillatory functional connectivity measures is favorable for gaining insight into normal vs. pathological brain function since cognitive processing is essentially dynamic.

To understand alterations within the DMN in TLE, we simultaneously recorded resting-state MEG and EEG data and then extracted MEG epochs off-line that were free of epileptic spikes and artifacts. Using a depth-weighted minimum norm estimate and imaginary coherence analysis, source-based functional connectivity in the DMN was compared between controls and patients with TLE. Additionally the laterality effect of TLE upon the DMN is investigated. This work provides insight into the novel neural underpinnings of the electrophysiological characteristics of the DMN in TLE.

## Methods

### 2.1. Subjects

Sixteen patients with medically intractable TLE (mean age: 27.8 years, 6 females, 1 left-handed) participated in this study. Subjects underwent a clinical evaluation in the Taipei Veterans General Hospital, including intensive video-scalp EEG monitoring, magnetic resonance (MR) imaging, and neuropsychological assessment. TLE was defined by the clinical seizure semiology of temporal lobe onset, presence of maximal epileptic discharges on anterior temporal electrodes (F7, F8, Ch1, or Ch2) or over the middle or posterior temporal electrodes (T3–T5, T4–T6) from interictal or ictal EEG data [29]. The laterality of epileptogenic focus was based on clinical history, interictal and ictal EEG recordings, and neuroimaging. Seizure types were documented according to the International League Against Epilepsy classification (Commission on Classification and Terminology of the International League Against Epilepsy, 1989). The clinical profile of the TLE patients is summarized in Table 1. The duration of epilepsy in the right TLE patients was significantly shorter than in the left TLE patients (p = 0.017). All included patients underwent MEG and EEG recordings.

**Table 1:** Clinical characteristic of TLE patients.

Patient	Sex	Type	Duration (year)	Frequency (/month)	MRI lesion	No. of AEDs
**Right TLE (n = 7)**						
1	M	CPS	5	<0.5	Right MTS	3
2	M	SGTCS	10	6	Normal	2
3	F	CPS	5	8	Right MTS	2
4	M	CPS	8	45	Normal	3
5	M	SPS	1	>60	VM, RMT	1
6	M	CPS	3	1	Tumor, RT	2
7	F	SPS	5	45	Encephalom, RT	5
**Left TLE (n = 9)**						
1	M	CPS	14	15	Left MTS	2
2	F	SGTCS	24	2.5	Normal	3
3	M	SGTCS	28	1.5	Tumor, F	4
4	F	CPS	22	6.5	Left MTS	4
5	M	CPS	17	2.5	Left MTS	3
6	F	SPS	14	15	Tumor, LT	2
7	M	CPS	6	5.5	Left MTS	2
8	M	SGTCS	4	0.5	Dysplasia, LMT	3
9	F	SPS	4	3.5	Encephalom, LT	3

R, right; L, left; T, temporal lobe; CPS, complex partial seizure

SPS, simple partial seizure; SGTCS, secondary generalized tonic-clonic seizure

MTS, mesial temporal sclerosis; VM, vascular malformation

Encephalom, encephalomalacia; F, frontal; AEDs, anti-epileptic drugs

Twenty-two healthy volunteers (mean age: 27.9 years, 9 females, 2 left-handed) were recruited as controls that matched the patients in age, gender, and handedness (all p > 0.05). None of the control subjects reported a history of symptoms of neurological or psychiatric disorders. Only MEG recordings were performed in control subjects. This study was approved by the Institutional Review Board of Taipei Veteran General Hospital. All participants gave written informed consent prior to any MEG or EEG recordings.

### 2.2. MEG and EEG recordings

MEG data were recorded using a whole-scalp 306-channel neuromagnetometer (Vectorview, Elekta Neuromag, Helsinki, Finland) composed of 102 identical triple sensor elements. Each sensor element consisted of one magnetometer and two orthogonal planar gradiometers. The exact position of the head with respect to the sensors was obtained by measuring magnetic signals produced by current leads to four head indicator coils at known sites on the scalp. Individual Cartesian coordinates were determined using a 3-D digitizer. The x-axis passed through the preauricular points from left to right, the y-axis passed through the nasion, and the z-axis pointed upward. In this study, data analysis was based on signals from the 204 planar gradiometers. For EEG recordings, 21 electrodes were positioned according to the International 10–20 System with the addition of bilateral cheek electrodes [29].

During the recordings, the subjects sat comfortably and kept awake with their head supported against the helmet of the neuromagnetometer. Any participant with excessive within-run head movement based on head position indicator (HPI) or who reported sleeping was re-run. Cortical spontaneous activities during eyes-closed and awake conditions were recorded for approximately 5 minutes with digitization rate 600 Hz. The MEG data from the resting state condition were extracted and fragmented into consecutive 2-s epochs in the off-line mode [42]. To eliminate interference caused by interictal spikes in TLE, the epochs of MEG and EEG data were visually examined off-line. For MEG spike detection in each epoch, superimposed MEG signals from 204 gradiometer channels were screened. Sharp signals that were clearly distinguishable from ongoing background activity were rejected and regarded as probable MEG spikes. In each epoch, EEG data were also used to identify spikes according to the definition of The International Federation of Societies for Electroencephalography and Clinical Neurophysiology (IFSECN) [43]. An epoch with a probable MEG or EEG spike was discarded in the present study.

In addition to spike elimination, we rejected epochs with sharp signals due to a clear contribution from heart beats, eye movements, or other physiological signals. For each subject, 30 epochs without spikes or artifacts were randomly selected for further analysis.

### 2.3. Data analysis

**Fig 1:**
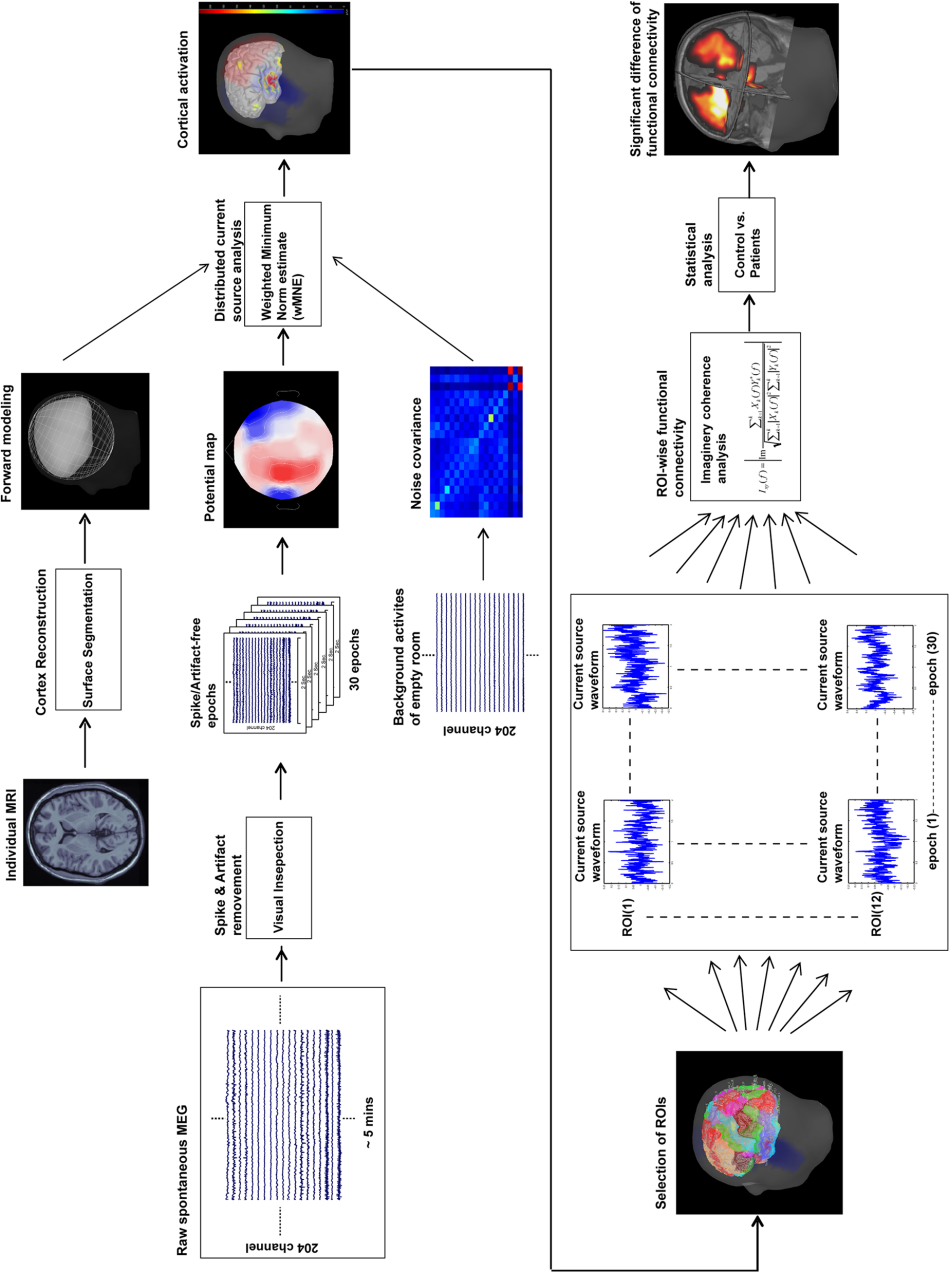
Data analysis procedure. The procedure for resting-state MEG data analysis that was used in this study is shown.

For the MEG data, the procedure used to analyze the resting-state functional connectivity analysis is shown in Fig 1. In addition to removing artifacts, two main analyses were performed. The first analysis was the minimum norm estimate to obtain the distributed source model of the MEG signals and the dynamic current strength of source activities in the DMN. The second analysis assessed source-based functional connectivity by calculating the imaginary coherence between two cortical sources among all epochs and compared the degree between two groups.

#### 2.3.1 Minimum norm estimate analysis

Depth-weighted minimum-norm estimation (MNE) was used to obtain the dynamics of cortical sources from the MEG data [44]. MNE offers fine spatial accuracy by using depth weighting [45] and is able to obtain simultaneous cortical sources that are distributed onto the brain surface [44, 46].

The details of our calculations of the forward model and inverse operator have been recently published [46]. An MRI-derived surface model of each participant's brain was automatically reconstructed by BrainVISA software (4.0.2, http://brainvisa.info) from the T1-weighted structural volumetric images. All images were acquired using a GE Discovery MR 750 3T (TR 9.4 msec, TE 4 msec, recording matrix 256 _256 pixels, field of view 256 mm, slice thickness 1 mm). The noise covariance was calculated from empty-room background activities which lasted ~30s in the beginning session of MEG recording. In the present analysis, the activation at each vertex (an equilateral triangle in the tessellation of the cortical surface) was estimated every 1.67 ms.

To measure the strength of cortical sources and calculate the functional connectivity in the common space across individuals, the reconstructed surface of each subject was morphed into the same source space, which was defined as the cortex from Colin27's anatomy. The morphing process first aligned the anterior commissure/posterior commissure axis of both cortical surfaces and then smoothed them to preserve only the main features. Next, the individual surface was deformed to match the MNI surface using an iterative closest point algorithm (BrainStorm Tutorials, http://neuroimage.usc.edu/brainstorm/Tutorials). Shepard’s method (weighted combination of a few nearest neighbors) was performed for the interpolation of the source strength from the subject surface to the Colin27 surface. The 12 regions of interest (ROIs) within the DMN included the posterior cingulate cortex (PCC), precuneus (PCu), inferior parietal cortex (IPC), medial temporal (MT), medial frontal cortex (MFC), and anterior cingulate cortex (ACC) in the bilateral hemispheres. The ROIs were selected from the cortical surface of default anatomy (MNI/Colin27) according to the automatic anatomical labeling template [47].

MNE presents each grid point of cortical source as a current dipole. The time series of the ROI was obtained from the averaged dynamic source strength across the current dipoles in the ROI. For averaged cortical activation measures, the current strength across time and epochs was calculated for each ROI within each subject. For functional connectivity analysis, the time-varying source strengths from each epoch were extracted for each ROI and subject. The MNE analysis was performed with Brainstorm [48], which is a documented program that is available for free download online under the GNU general public license (http://neuroimage.usc.edu/brainstorm).

#### 2.3.2 Source-based functional connectivity analysis

Imaginary coherence (IC) was used to estimate the functional connectivity to minimize any crosstalk effects between sources [49]. In addition, this technique has been suggested to effectively reveal altered functional connectivity in cases of brain lesions [42], brain tumors [50], and in AD patients [13] during a resting-state condition. IC rejects the spurious connectivity between two cortical sources with no time delay, which could be attributed to a common source or volume conduction. Thus, IC represents the interactions between brain regions with a specific time lag [49]. The IC was calculated according to the following formula:

$$I_{xy}\left(f\right)=\left|Im\frac{\sum_{k=1}^{K}X_{k}\left(f\right)Y_{k}^{*}\left(f\right)}{\sqrt{\sum_{k=1}^{K}{\left|X_{k}\left(f\right)\right|}^{2}\sum_{k=1}^{K}{\left|Y_{k}\left(f\right)\right|}^{2}}}\right|$$

where *I*_*xy*_ *(f)* is the IC between a given paired-ROIs for each frequency bin, *Im* is the imaginary part of the complex production, *X*_*k*_ and *Y*_*k*_ are the source-based spectrums from two ROIs (Hanning-windowed, Fourier transformed time-varying source strengths), * denotes the complex conjugate, and *K* is the number of 2-s epochs.

The calculation of IC was programmed using Matlab computing software (The Math Works, Natick, MA), which could be freely downloaded on Google Drive (https://drive.google.com/folderview?id=0B6YoD6Poj9GEfkVyWTliR0tlMHByMjdzS291ME91XzBvaTFmNEx0SjhXLXB4TUJRbVZ4TE0&usp=sharing). The frequency bands of the IC were classified into delta (1–4 Hz), theta (4–8 Hz), alpha (8–13 Hz), beta (13–25 Hz), and gamma (25–40 Hz). The IC values for each frequency band were calculated from the average of 30 epochs. The IC values for every subject were computed for all ROIs and the full 12×12 adjacency matrix was estimated. The mean IC value, which is computed from the average of the IC values between one ROI and the other 11 ROIs (average of 11 paired IC values), denotes the mean functional connectivity within the DMN for each of the 12 ROIs.

#### 2.3.3 Statistical analysis

An independent t-test was performed to examine significant differences between groups (TLE and control) in the averaged cortical activation within each ROI. The difference between groups with respect to the mean IC values was tested in each frequency band. The Bonferroni correction for multiple comparisons was conducted on the IC values with the factors of ROI and frequency band. The IC values in the left TLE and the right TLE were compared statistically to controls using the Wilcoxon rank-sum test. Statistical analyses were performed using Matlab toolbox (version 7.10, R2010b, http://www.mathworks.com/) or the SPSS software package (SPSS Inc, Chicago). A p-value < 0.05 was considered statistically significant.

## Results

### 3.1 Cortical activation measurements

**Fig 2:**
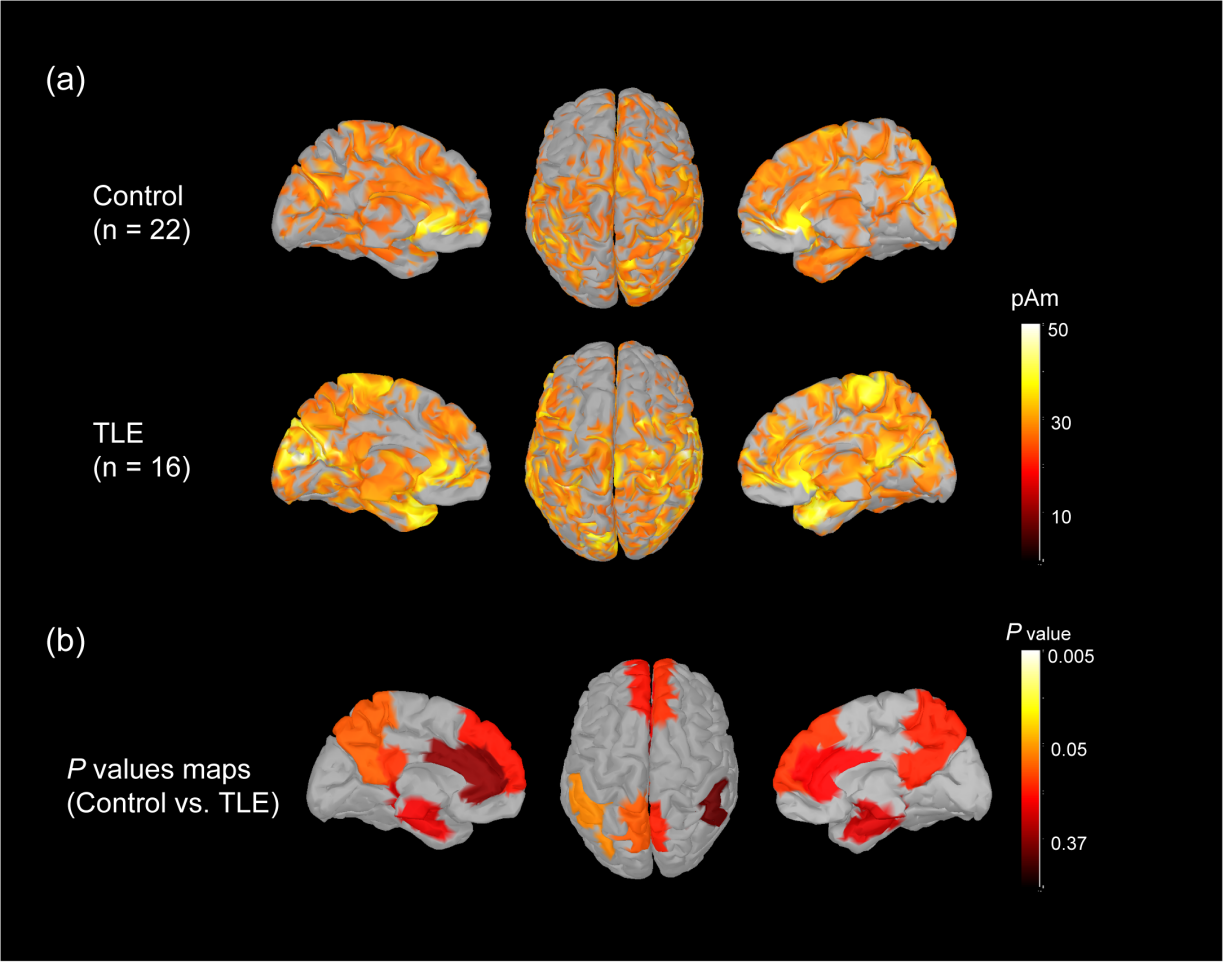
Cortical activation during resting state condition. (a) The distribution of the average cortical activation during an eyes-closed resting-state condition in 22 control subjects and 16 TLE patients. The activation maps are shown from the top and medial views. The source values were smoothed after re-interpolation (the size of the smoothing kernel = 5). Only the cortical sources which strength is larger than 60% of maximal value are displayed. The current strength for cortical sources shown is color coded with large values represented in white. (b) The p-value maps from the activation comparisons between control subjects and TLE patients are shown. The maps are color coded with significance represented in white and light yellow.

We calculated the grand-averaged activation on cortical surfaces with top and medial views of both hemispheres across the controls and TLE subjects. The activation maps over the frontal, parietal, temporal, occipital and midline regions were similar between the two groups (Fig 2(A)). The normalized source strength of underlying cortical sources was color coded. The normalized averaged strengths of 12 ROIs within the DMN showed no significant differences between the two groups (all p > 0.05). As shown in Fig 2(B), the color-coded maps display the p values for each ROI.

### 3.2 The mean functional connectivity within the DMN

**Fig 3:**
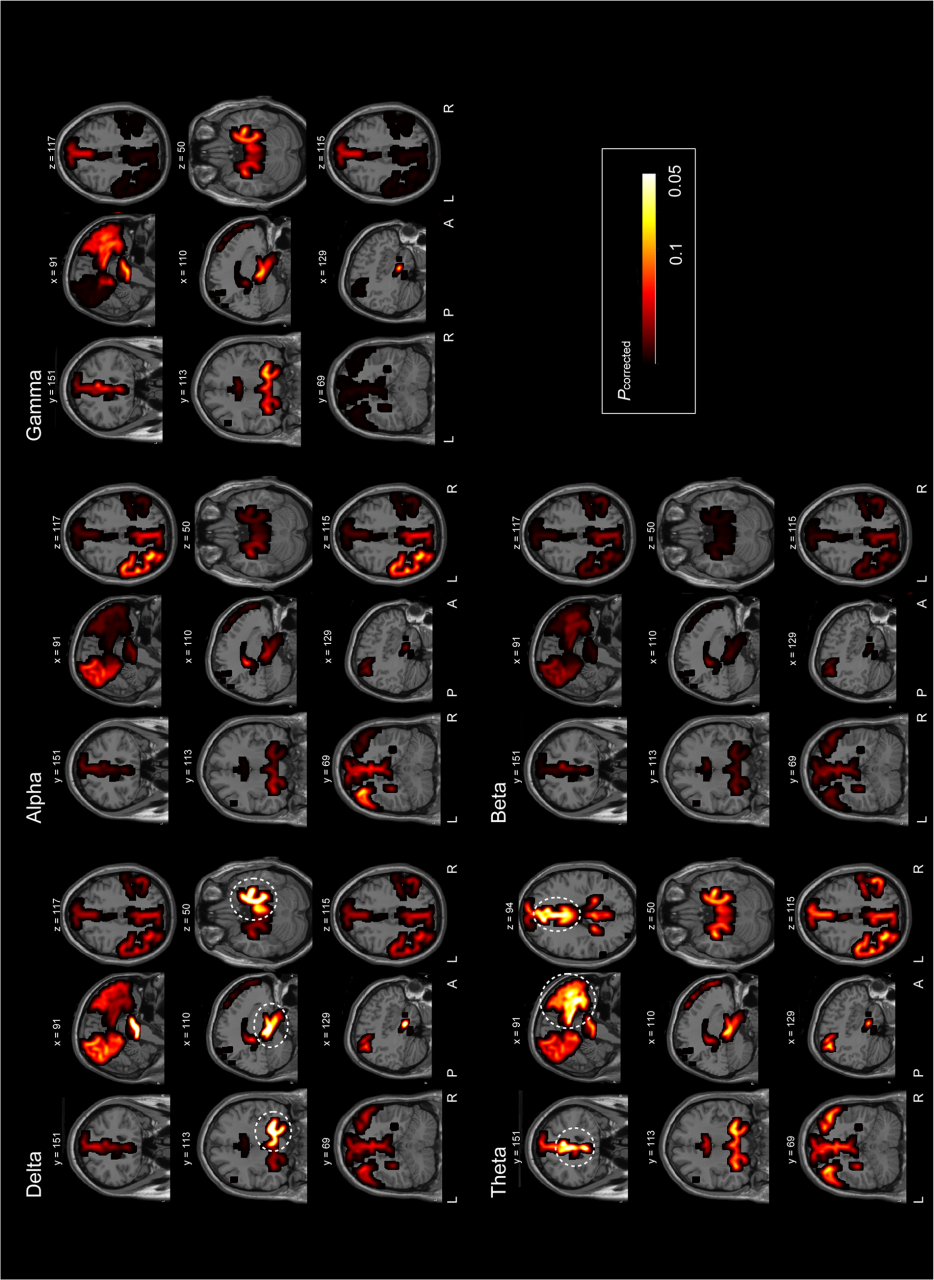
Difference of functional connectivity between control subjects and TLE patients. The p-value maps on the sagittal, coronal and axial MR images show the significant differences in mean functional connectivity within the DMN between control subjects and TLE patients in the delta, theta, alpha, beta and gamma bands. Cortical areas encircled by dashed circles indicate cortical areas with significant changes. The maps are color coded with significant values (p < 0.05) denoted in white. The number above the image indicates the slice number.

For each frequency band, distinct mean IC values (within the DMN) were compared between TLE patients and controls (Fig 3, on the MRIs; S1 Fig, on the cortical surfaces). The corrected p values for each ROI in each frequency band were mapped onto the coronal, sagittal, and axial MR images. In the delta band, a significantly larger mean IC value was found in the right MT in TLE patients when compared with control subjects (corrected p = 0.006, TLE = 0.144±0.001, controls = 0.133±0.002). Moreover, in the theta band, the mean IC value was significantly larger in the bilateral ACC of TLE patients when compared with control subjects (left ACC: corrected p = 0.026, TLE = 0.197±0.002, controls = 0.186±0.002; right ACC: corrected p = 0.037, TLE = 0.195±0.002, controls = 0.184±0.002). No significant differences between groups were observed for each ROI with respect to the alpha, beta, and gamma bands (all p > 0.05).

### 3.3 The effect of laterality on the mean functional connectivity within the DMN

**Fig 4:**
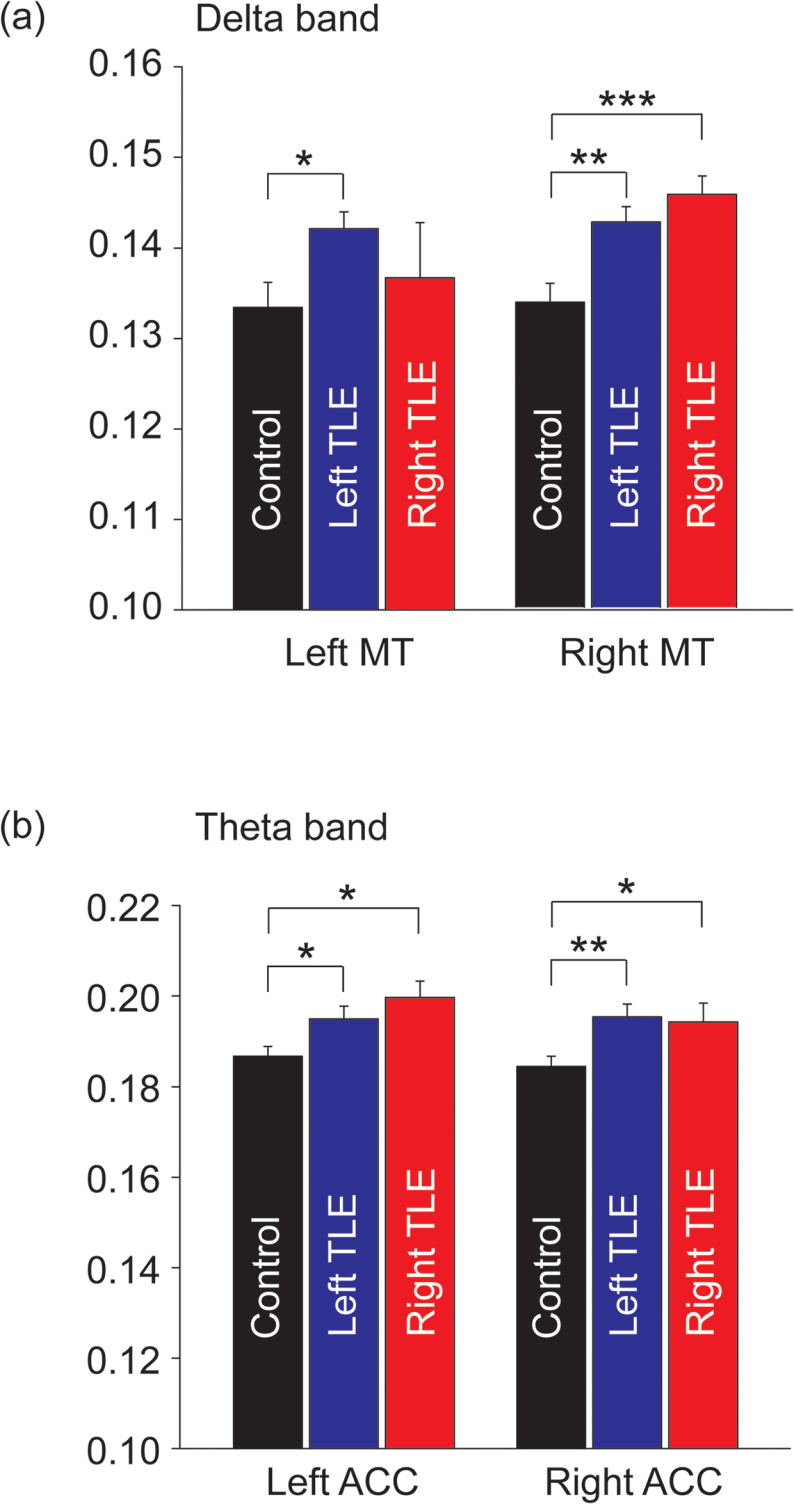
Bar plots for the laterality effect on functional connectivity. (a) The difference in the delta functional connectivity in the left and right MT regions between controls, and left and right TLE patients is shown. (b) There were significant differences in the theta functional connectivity in the left and right ACC regions between controls, and left and right TLE patients. Each error bar represents the *standard error of the mean* (*SEM*). * p < 0.05; ** p < 0.01; *** p < 0.001.

To determine the effect of lateralized TLE on the DMN, the mean IC values for patients were compared with controls (Fig 4). We found that the left TLE patients showed a larger mean IC value in the delta band in the bilateral MT when compared with controls (all p < 0.05). For right TLE patients, an increased mean delta IC value was only found in the right MT (p < 0.001). For both left and right TLE patients, a larger mean IC value at theta band in the bilateral ACC was found when compared with controls (all p < 0.05).

## Discussion

During interictal spike-free periods, an alteration of resting-state functional connectivity within the DMN was observed in TLE patients. This was shown by increases in the mean functional connectivity at specific rhythms. In TLE patients, there was an increased mean IC values in the right MT region at delta band (TLE: 0.1439±0.0013, controls: 0.1337±0.002) and in the bilateral ACC region at theta band (left ACC, TLE: 0.197±0.0022, controls: 0.1867±0.0021; right ACC, TLE: 0.1949±0.0023, controls: 0.1844±0.0023). With regard to the effect of lateralized TLE, asymmetric increased delta connectivity with right MT was found in right TLE patients, while symmetric increased theta connectivity with bilateral ACC was observed in left and right TLE patients.

### 4.1 Cortical activation in the DMN

A similar activation pattern within the DMN was found between controls and TLE patients. Previous TLE studies have reported that the brain regions within the DMN may be decreased or increased BOLD responses in fMRI [27, 51, 52], enhanced neural activities in MEG [28], and high gamma power increase in intracranial EEG [41, 53]. These phenomena may be the effect or spreading of epileptic spikes from the epileptogenic zone [19, 52]. In the present study, no significant difference of the DMN activation between TLE patients and controls suggests that the resting-state MEG epochs with our spike elimination may be notably free of epileptic activities. Moreover, the activation of DMN was thought to support consciousness [4]. The level of consciousness during resting state recording could be similar between groups.

### 4.2 The alterations of mean functional connectivity in TLE patients

Without the contamination of interictal spike activities, increased functional connectivity in TLE patients was found in the right MT region at delta band and in the bilateral ACC regions at theta band. An increased level of functional connectivity during a resting-state condition was reported in various types of epilepsy in the EEG [54, 55], MEG [56], and fMRI studies [57–64]. These evidences are in line with the consistent morphological changes involved in distinct brain regions in TLE patients [65], or between and within hemispheres in epilepsy [66], which was attributed to an altered configuration of grey matter regions and interconnecting white matter tracts in TLE [23]. Moreover, it has been suggested that an enhanced connection may be the consequence of functional reorganization or plasticity [57, 60, 64] and cortical inhibitory mechanisms [56] in epilepsy. Apart from an enhanced connectivity, the association between disruption of cortical connections in the DMN and cognitive impairment of epilepsy patients was noted in previous fMRI studies [23, 57, 62, 66, 67]. This discrepancy of the DMN alterations might stem from (1) the fundamental methodological difference between MEG and fMRI [36, 37], (2) the frequency range studied, or (3) the effect of interictal spike activities [27, 51, 52]. Of note, previous morphometric analysis showed a bilateral increase in gyral complexity in TLE patients [68], which represented the increase of fiber connectivity. Luo and colleagues [57] speculated that increased cortical connectivity may reflect the chronic, abnormal, functional integration in the brain in epilepsy. We suggest that reinforced links within the DMN characterize the cortical alteration during resting state condition in TLE patients. It could be related to maintain the brain functioning or facilitate the formation of the epileptogenic network.

### 4.3 The effect of lateralized temporal lobe epilepsy on the functional connectivity of the DMN

A larger mean connectivity in the right MT region at delta band is consistent with prior studies that found abnormal hub properties or strongly connected network nodes in the epileptogenic regions [61, 66, 69]. Notably, the left TLE patients showed enhanced connectivity in the bilateral MT regions, whereas the right TLE patients had an increased connectivity only in the right MT region. Bilateral hemispheric alterations of cortical activation or functional connectivity in TLE patients could be the consequence of unilateral damage in the MT region [25, 70, 71]. It suggests that although no structural change in the MT region contralateral to the seizure focus was observed, functional brain plasticity could occur in the bilateral MT regions [25].

Previous studies found that the side of seizure onset has distinct effect on the connectivity and structural abnormalities in TLE [72, 73]. In this study, the asymmetric connectivity increase in the right TLE patients may be due to a difference in cortical plasticity patterns between the left and the right TLE patients [25]. Another possibility is that the contralateral compensatory mechanism was correlated with the duration of epilepsy [21, 60, 61]; therefore, the contralateral compensation in the right TLE patients with shorter epilepsy duration in this study remained deficient. The increased connectivity pattern in the right MT region for both the right and left TLE patients suggests that this region may act as a vigorously connected node even during spike-free resting-state condition.

### 4.4 The oscillatory characteristics of the altered functional connectivity in TLE patients

Delta activity is a neuropathological oscillation of the brain [74–78]. Increases in delta connectivity was related to the loss of intracortical inhibition [79–81] and an abnormal cortical hypercoupled state [54, 82, 83]. During interictal period without spikes or ictal discharges, enhanced delta connectivity is consistent with a prior EEG study [83]. It may indicate that the resting-state dynamics in a reorganized network for TLE are synchronized at delta band as the hub in the right MT region.

In the bilateral ACC regions, enhanced theta connectivity was observed in the right and the left TLE patients. This result is concordant with the altered ACC connectivity in epilepsy in the previous studies [54, 60–62]. The ACC region has been shown as a predominant hub that might be activated during affective, cognitive and motor tasks requiring attention in the healthy brain [84–87], and it might be involved in the spreading and generalization of epileptic discharge and the thalamocortical network in the epilepsy patients [88, 89]. Moreover, frontal theta activity was associated with the cortical processes of working memory [90], associative learning, attention and integrative cognition and association [91]. We speculate that the altered connectivity in the bilateral ACC at theta band might underlie the neurophysiological mechanisms of cognitive processes and epileptogenic network in TLE patients.

### 4.5 Limitations

With regard to the methodology of resting state MEG analysis, the preprocessing adapted in the present study simply identified and removed the data epochs with artifacts through visual inspection or automatic detection, which is one of the two general strategies for dealing with artifacts in MEG [92], without the independent component analysis (ICA) or signal space projection (SSP) methods. This preprocessing method in resting-state MEG studies has been reported that the functional connectivity between cortical regions could be well measured [93, 94]. Moreover, the nature of imaginary coherence analysis could be robust to spurious connectivity caused by linear signal leakage [95, 96]. It is of note that Gross and coauthors [92] have not concluded the priority of all the above artifact correction methods, since the effect of artifact correction methods on resting state MEG analysis still needs to further studies to clarify.

In the present study, if the responses were contaminated with strong ECG artifacts and clean responses could not be obtained through visual inspection or automatic detection, we would set 8–12 lateral temporal channels as bad channels. Of course they were ignored from the source estimation. The effect of removed channels on the minimum norm solution depends on their number and the positions in relation to individual’s brain and the regions of interest. In the present study, it is hard to answer the true impact of bad channels on functional connectivity measures, even if no significant difference of cortical activation between groups was found. However, the impact may be negligible due to few subjects with strong ECG contaminations in this study (controls: 3/22; patients: 2/16).

In this study, TLE patients with distinct brain lesions and seizure types were included. Although the altered electrophysiological connectivity patterns for a specific symptom type or lesion may be of concern, the present findings from the heterogeneity of the TLE patients may provide the common underlying electrophysiological mechanism. Further studies are needed to confirm our present results and extend the investigation to include larger populations. Moreover, our experimental design did not control the confounding effects from anti-epileptic drugs (AED). However, the effect of AED on connectivity remains unresolved [21]. A longitudinal follow-up study is needed to elucidate the impact of AED upon the DMN connectivity.

## Conclusion

Independent of interictal or ictal spike activities, TLE patients showed aberrant DMN connectivity, especially with the MT and ACC regions at delta and theta bands. These findings suggest that there are reinforced links to maintain brain functioning or form the epileptogenic network. These findings provide important functional and electrophysiological results related to the pathological mechanism of TLE. Further investigation of the association between the intrinsic neural network changes and the progression of disease or cognitive dysfunction in TLE patients is warranted.

## Supporting Information

S1 Fig Difference of functional connectivity plotted on the cortical surfaces. The p-value maps on the cortical surfaces with medial and top views exhibit the differences of functional connectivity between control subjects and TLE patients in the delta, theta, alpha, beta and gamma bands. Cortical areas encircled by dashed circles indicate cortical areas with significant changes. A, anterior; P, posterior; L, left; R, right.
(TIF)
