# Supplementary figures and images for: Increased Intrinsic Connectivity of the Default Mode Network in Temporal Lobe Epilepsy: Evidence from Resting-State MEG Recordings
Source: PLoS One. 2015 Jun 2;10(6):e0128787. doi: 10.1371/journal.pone.0128787 (PMC4452781; doi:10.1371/journal.pone.0128787)

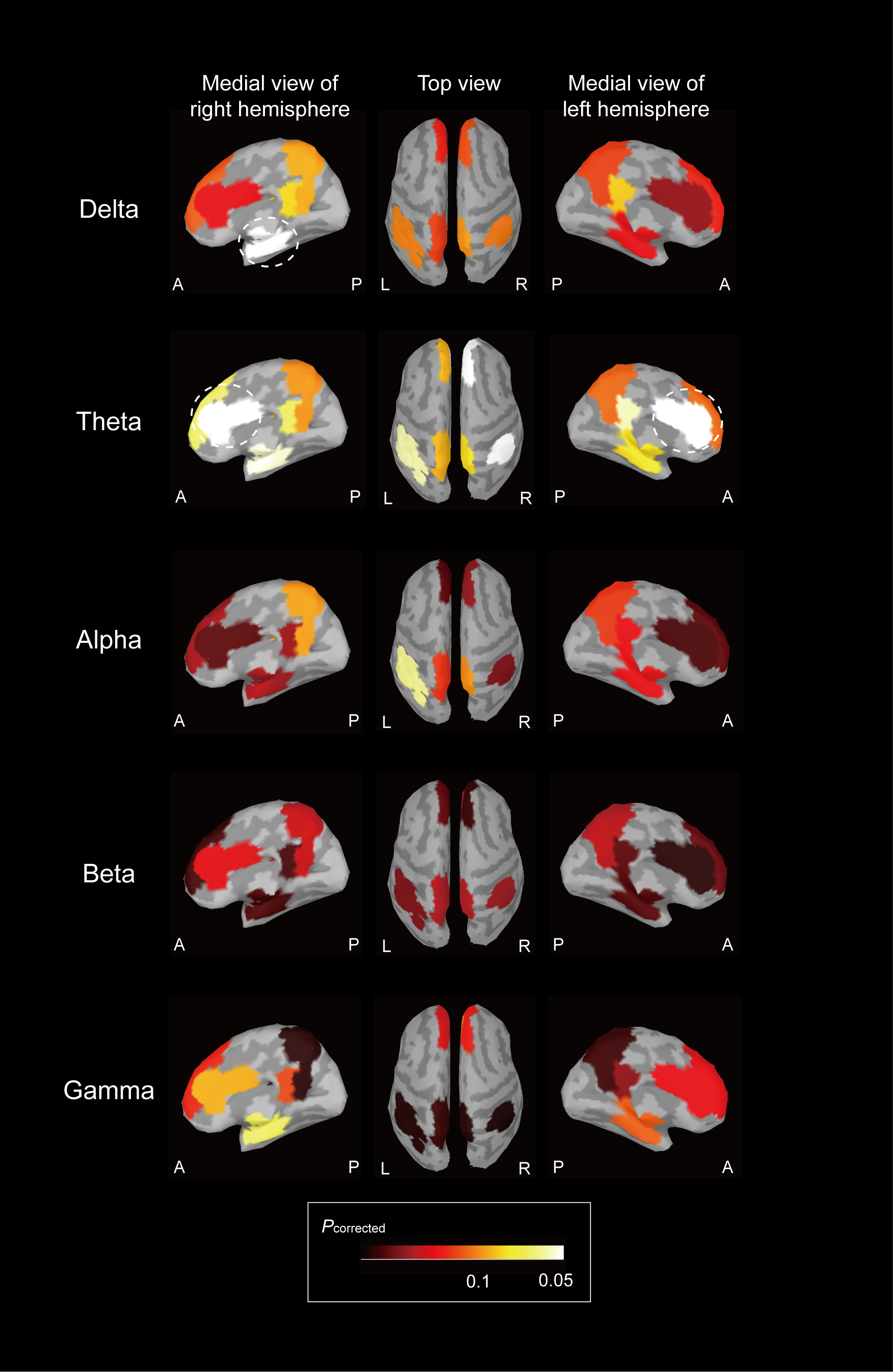

Supplement: S1 Fig — The p-value maps on the cortical surfaces with medial and top views exhibit the differences of functional connectivity between control subjects and TLE patients in the delta, theta, alpha, beta and gamma bands. Cortical areas encircled by dashed circles indicate cortical areas with significant changes. A, anterior; P, posterior; L, left; R, right. (TIF) [file pone.0128787.s001.tif]
